# Four-Octyl Itaconate Protects Chondrocytes against H_2_O_2_-Induced Oxidative Injury and Attenuates Osteoarthritis Progression by Activating Nrf2 Signaling

**DOI:** 10.1155/2022/2206167

**Published:** 2022-01-27

**Authors:** Peng Zhang, Xiaotong Wang, Qiliang Peng, Yesheng Jin, Gaolong Shi, Zhihai Fan, Zhiqiang Zhou

**Affiliations:** ^1^Department of Orthopedics, The Second Affiliated Hospital of Soochow University, Suzhou 215000, China; ^2^Department of Hepatology and Gastroenterology, The Affiliated Infectious Hospital of Soochow University, Suzhou 215000, China; ^3^Department of Radiotherapy & Oncology, The Second Affiliated Hospital of Soochow University, Suzhou 215000, China; ^4^Department of Orthopedics, Wuxi Ninth People's Hospital Affiliated to Soochow University, Wuxi, Jiangsu 214026, China

## Abstract

Nrf2 is a critical regulator of the antioxidant defense systems in cellular protection. Emerging evidence has shown that four-octyl itaconate (OI) activates Nrf2 cascade. In this study, the chondroprotective effects of OI on H_2_O_2_-stimulated chondrocytes and DMM-induced osteoarthritis (OA) progression were investigated. In primary murine chondrocytes, OI interrupted the binding of Keap1 and Nrf2, leading to accumulation and nuclear translocation of Nrf2 protein, as well as transcription and expression of Nrf2-dependent genes, such as *HO-1*, *NQO1*, and *GCLC*. Furthermore, OI inhibited cell death and apoptosis, as well as H_2_O_2_-stimulated ROS generation, lipid peroxidation, superoxide accumulation, and mitochondrial depolarization in chondrocytes, which were abolished by the silence or depletion of Nrf2. In addition, *in vivo* experiments revealed the therapeutic effects of OI on OA progression in a DMM mouse model. Collectively, these results suggested that OI might serve as a potential treatment for OA progression.

## 1. Introduction

Osteoarthritis (OA), featured by progressive cartilage degradation, is one of the most prevailing degenerative joint disorders among the elderly, leading to severe pain and joint dysfunction [1, 2]. Studies have shown that oxidative stress caused by sustained reactive oxygen species (ROS) production is the major reason for chondrocyte apoptosis, eventually leading to OA pathogenesis [3]. In contrast, clearance of ROS offers significant chondrocyte protection against oxidative injury.

Nuclear factor E2-related factor 2 (Nrf2) is a principal mediator of the antioxidant defense systems in cellular protection [4–7]. Under homeostatic circumstances, Nrf2 associates with Kelch-like ECH-associated protein 1 (Keap1) and localizes in cytosol [5, 6, 8, 9]. Under oxidative stress, Keap1 is modified, thus leading to separation of Nrf2 from Keap1, as well as Nrf2 stabilization, accumulation, and translocation into the nucleus. Then, Nrf2 associates with antioxidant response elements (AREs) and triggers the transcription and expression of a series of Nrf2-dependent genes [10, 11], including *heme oxygenase 1 (HO1)*, *NAD (P) H quinone oxidoreductase-1 (NQO1)*, and *γ-glutamyl cysteine ligase catalytic subunit (GCLC)*, and all of them exhibit robust antioxidant effects to protect cells [5, 12].

Previous researches have confirmed that itaconate could activate Nrf2 pathway [13, 14]. Specifically, itaconate can modify Keap1, leading to separation of Nrf2 from Keap1, followed by Nrf2 stabilization, accumulation, and activation. Additionally, several studies have shown that the itaconate derivative four-octyl itaconate (OI) potently exerts cytoprotection against oxidative injury via activating Nrf2 signaling [13, 15–17]. For example, Tang et al. revealed OI stimulated Keap1-Nrf2 pathway to efficiently downregulate the generation of proinflammatory cytokines in macrophages [16]. Another study revealed that OI induced Keap1-Nrf2 cascade to protect neurons against hydrogen peroxide (H_2_O_2_) [17]. Moreover, a recent study revealed that OI can protect osteoblasts against H_2_O_2_ via activating Nrf2 pathway [18]. However, the underlying effect of OI on chondrocytes remains to be fully elucidated. The purpose of this research was to test whether OI could exert chondrocyte protection against oxidative injury and explore the possible mechanism. In addition, the therapeutic value of OI in OA mouse model was also evaluated *in vivo*.

## 2. Materials and Methods

### 2.1. Chemicals, Reagents, and Antibodies

The antibodies of this research were provided by Cell Signaling Technology (Shanghai, China). JC-1 dye, carboxy-H2DCFDA dye, TRIzol, lipofectamine 2000, Annexin V, and propidium iodide (PI) were purchased from Invitrogen (Shanghai, China). Puromycin, polybrene, caspase-3 assay kit, MG-132, cycloheximide (CHX), and cell culture reagents were obtained from Sigma-Aldrich (St. Louis, MO, USA). ELISA kit was provided by Roche (Shanghai, China). All reagents for cell culture were purchased from Gibco (Suzhou, China).

### 2.2. Western Blotting

Western blotting assays were carried out as previously reported [19, 20]. In brief, total proteins were extracted from chondrocytes and isolated in RIPA lysis buffer. Then, 40 *μ*g of total proteins were loaded into well on sodium dodecyl sulphate-polyacrylamide gel electrophoresis (SDS-PAGE) and transferred to a polyvinylidene difluoride (PVDF) membrane (Bio-Rad, USA). Next, the membranes were blocked with 5% skim milk, followed by incubation with primary antibodies against GAPDH, Tubulin, CypD, p53, ANT-1, Lamin B1, Keap1, Nrf2, HO1, NQO1, and GCLC. Antibodies were all used at 1 : 1,000 dilution unless otherwise indicated. Subsequently, the membranes were incubated with the relevant secondary antibody, and the protein bands were visualized by an enhanced chemiluminescence kit, with ImageJ software used to quantify the band intensity.

### 2.3. Real-Time PCR

After treatment, total RNA was isolated from chondrocytes with TRIzol reagents (Invitrogen). Next, cDNA was prepared with 1 mg of total RNA (MBI Fermantas, Germany). The thermocycler settings were 95°C for 10 min, then 95°C for 15 s, and 60°C for 1 min, conducted for 40 cycles. The process was performed via the CFX96 Real-Time PCR system (Bio-Rad, USA). The cycle threshold (CT) value was obtained and standardized to GAPDH levels. The relative mRNA levels to each target gene were measured via the 2^−∆∆*C*t^ approach. The mRNA primers for the listed genes were obtained from Dr. Di [17].

### 2.4. Nrf2 shRNA

Chondrocytes were transfected with two lentiviral Nrf2 shRNAs (Santa Cruz Biotechnology, sc-37030-V/“shNrf2-a” and sc-44332-V/“shNrf2-b”). After 24 h, stable chondrocytes were selected by puromycin for 12 days. Finally, the transfection efficiency was detected by western blotting and qPCR analyses.

### 2.5. CRISPR-Cas9-Mediated Gene Knockout (KO)

Primary murine chondrocytes were transfected with a CRISPR/Cas9-Nrf2-KO-GFP-puro construct or a CRISPR/Cas9-Keap1-KO-GFP-puro construct, purchased from Santa Cruz Biotechnology. A FACS-mediated selection of GFP-positive cells was performed. And single cells were cultured in a 96-well plate to generate monoclonal stable cells. Nrf2 KO or Keap1 KO were verified by western blotting and qPCR analyses.

### 2.6. Mitochondrial Depolarization

Following H_2_O_2_ stimulation, mitochondrial depolarization (“∆*Ψ*”) in chondrocytes was determined by the mitochondrial targeting dye JC-1, which forms green monomers with mitochondrial depolarization [21]. The method for the JC-1 assay has been reported previously [22].

### 2.7. Co-Immunoprecipitation (co-IP)

The detailed Co-IP protocol has been reported elsewhere [23]. In brief, total cell lysates were precleared with protein A/G beads (Sigma), followed by Keap1 antibody incubation overnight. The Keap1-immunoprecipitated proteins were captured by protein A/G beads and measured by western blotting analyses.

### 2.8. Mitochondrial Immunoprecipitation (Mito-IP)

The detailed Mito-IP method has been described elsewhere [24].

### 2.9. ROS Detection

ROS content was determined following an earlier described method [25]. In brief, chondrocytes were incubated with 1 *μ*M of carboxy-H2DCFDA, followed by detection of the DCF fluorescence signal at a wavelength of 550 nm with a fluorescence spectrofluorometer (Thermo Scientific, China).

### 2.10. Lipid Peroxidation

As reported previously [25], the level of cellular lipid peroxidation was determined by thiobarbituric acid reactive substances (TBAR) activity.

### 2.11. Superoxide Detection

The level of cellular superoxide was detected by the superoxide colorimetric assay kit (Beyotime, Wuhan, China). Briefly, treated chondrocytes were incubated with superoxide detection reagent, followed by test of the superoxide absorbance at a wavelength of 450 nm.

### 2.12. Single Strand DNA (ssDNA)

The level of cellular ssDNA was determined following the previously reported method [22].

### 2.13. Primary Cultivation of Mouse Chondrocytes

Immature C57BL/6 mice were provided from the Animal Center of Chinese Academy of Sciences (Shanghai, China) and euthanized with excess pentobarbital sodium. First, articular cartilage tissues from the knee joints were isolated. Next, the collected cartilage was washed with PBS 2 to 3 times, shredded, digested by 0.1% collagenase II for 4 h at 37°C, and centrifuged. After resuspension in PBS, the cartilage was incubated in DMEM/F12 added with 10% fetal bovine serum and 1% penicillin/streptomycin at 37°C with 5% CO_2_. After the chondrocytes reached 80% ~90% confluency, 0.25% trypsin EDTA solution was utilized for cell passage, and cells of passages 1 to 3 were employed in the study.

### 2.14. Cell Viability

A cell counting kit-8 (CCK-8, Dojindo Co., Kumamoto, Japan) was employed to determine the cell viability in accordance with the manufacturer's guidelines. Initially, primary murine chondrocytes were incubated into 96-well plates (3000 cells each well) for 12 h, subsequently added with OI for 48 h. After each well was washed with PBS, a 100 *μ*l DMEM/F12 containing the CCK-8 reagent was added and incubated at 37°C for 2 h, followed by the calculation of the optical density (OD) value at a wavelength of 450 nm.

### 2.15. TUNEL Assay

Chondrocytes were originally seeded into six-well tissue culture plates at 30,000 cells/cm^2^. Then, the nuclei were stained with TUNEL reagent (5 *μ*M, Sigma) and DAPI (1 *μ*M, Sigma) for 30 min in the dark. The TUNEL ratio (TUNEL/DAPI × 100%) was calculated.

### 2.16. Animals and Treatment

Male C57BL/6 mice aged 8 weeks were provided from Shanghai Animal Center of the Chinese Academy of Sciences and housed in a specific pathogen-free (SPF) facility. All animal procedures were performed according to the Guidelines for the Care and Use of Laboratory Animals of the National Institutes of Health and approved by the Institutional Animal Care and Use Committee of Soochow University (No. ECSU-2020000191). As mentioned previously [26], a mouse model of OA was established by surgical destabilization of the medial meniscus (DMM). Then, the mice (*n* = 15) were randomly classified into three groups of five mice each: sham group, DMM + saline group, and DMM + OI group, respectively. Mice in the DMM + OI group were intraperitoneally administered OI at doses of 25 mg/kg/day in (2-hydroxypropyl)-*β*-cyclodextrin diluted in PBS or vehicle control for 8 weeks. The control mice received an equivalent volume of saline instead. After eight weeks, the cartilage tissue samples were collected.

### 2.17. Statistical Analysis

All experiments were conducted three times. The values are represented as mean ± standard deviation (SD). SPSS 20.0 was utilized for statistical analyses. Data were processed by one-way analysis of variance (ANOVA), and the Tukey test was used for comparisons between groups. Nonparametric data (OARSI scores) were analyzed by the Kruskal–Wallis H test. A *P* value of less than 0.05 is considered to be significant.

## 3. Results

### 3.1. OI Activated Nrf2 Signaling in Primary Murine Chondrocytes

**Figure 1:**
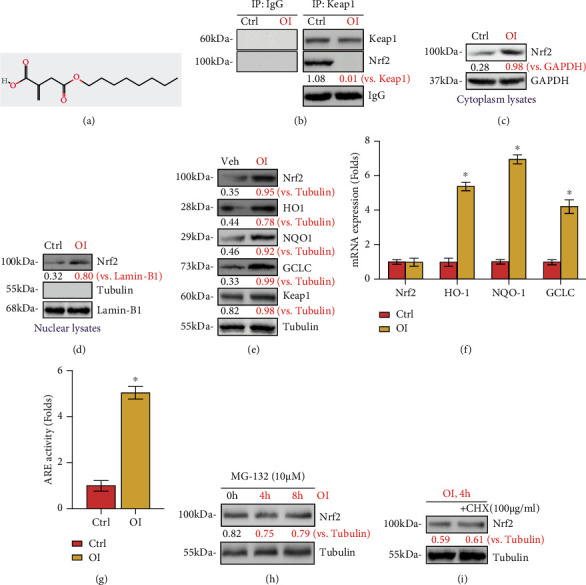
OI activated Nrf2 signaling in primary murine chondrocytes. (a) The chemical structure of OI. Primary murine chondrocytes were added with OI (25 *μ*M) or vehicle control (“Veh”). (b) Coimmunoprecipitation (“co-IP”) assay was employed to examine the binding of Keap1 and Nrf2. The expressions of the indicated proteins in total cellular lysates (c) and nuclear lysates (d) were determined by western blotting. (e, f) Protein and mRNA expressions of the listed genes were investigated by western blotting analyses and qPCR; (g) relative ARE luciferase activity was detected. Primary murine chondrocytes were added with MG-132 (10 *μ*M, 24 h) and cycloheximide (CHX, 100 *μ*g/ml, 12 h), and OI (25 *μ*M, 4 h) was given subsequently. (h, i) Nrf2 and tubulin protein expressions in total cellular lysates were examined. The values are represented as mean ± SD. ∗*P* < 0.05 vs. “Veh” group.

The potential effect of OI on the Nrf2 cascade in cultured primary murine chondrocytes was examined. The results of coimmunoprecipitation (co-IP) assays (Figures 1(b) and 1(c)) revealed that Keap1 immunoprecipitated with Nrf2, and the connection between Keap1 and Nrf2 was disrupted by OI, with the concentration of 25 *μ*M based on previous researches [17, 18]. Then, Nrf2 protein was stabilized and accumulated in chondrocyte cytosol with OI treatment, while the Keap1 protein remained unchanged. In addition, the results demonstrated that Nrf2 translocated into the nucleus, evidenced by the potently elevated Nrf2 protein levels in the nuclei determined by western blotting analysis (Figure 1(d)). Notably, the protein (Figure 1(e)) and mRNA (Figure 1(f)) levels of Nrf2-ARE target genes (*HO1*, *NQO1*, and *GCLC*), as well as those of the ARE luciferase activity (Figure 1(g)), were robustly increased in OI-treated chondrocytes, with unchanged Nrf2 mRNA levels (Figure 1(f)) and Keap1 expression (Figure 1(d)). More importantly, the Nrf2 protein level was not significantly upregulated by OI in chondrocytes treated with MG-132, an established cell-permeable proteasome inhibitor (Figure 1(h)). Furthermore, the Nrf2 protein level was not distinctly affected by OI in chondrocytes after treatment with cycloheximide (CHX), a well-known protein synthesis inhibitor (Figure 1(i)). These results suggested that OI-induced Nrf2 protein augmentation was not the result of protein synthesis but protein stabilization. Taken together, the results suggested that the Nrf2 cascade was activated by OI in primary murine chondrocytes.

### 3.2. OI Inhibited H_2_O_2_-Stimulated Cell Death and Apoptosis in Primary Murine Chondrocytes

**Figure 2:**
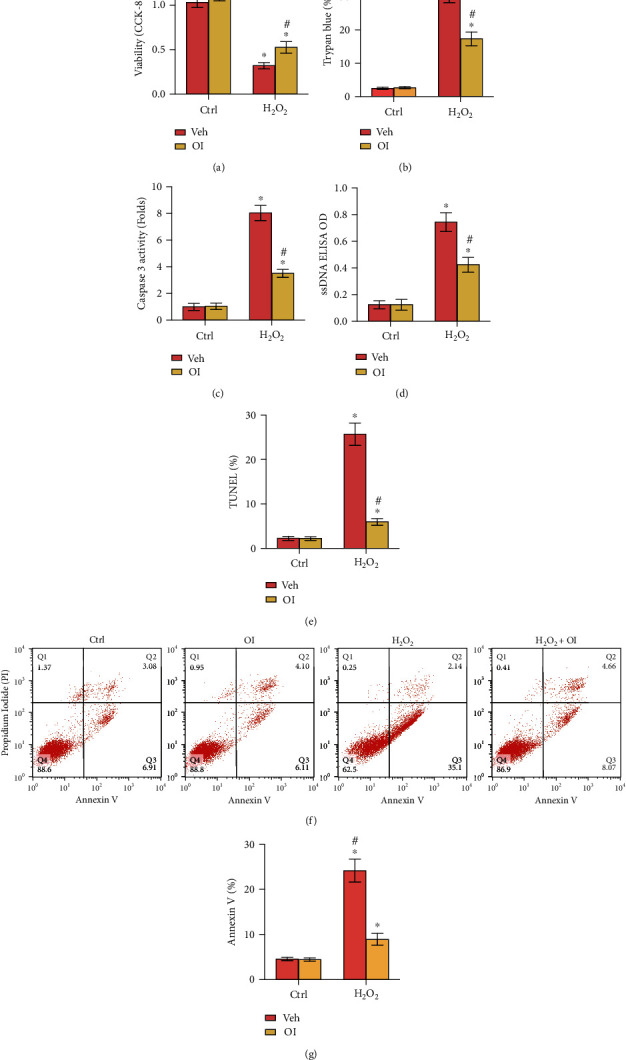
OI inhibited H_2_O_2_-stimulated cell death and apoptosis in primary murine chondrocytes. Primary murine chondrocytes were added with OI (25 *μ*M) or vehicle control (“Veh”), followed by H_2_O_2_ (300 *μ*M) stimulation. Cell viability (a), cell death (b), caspase-3 activity (c), accumulation of single-stranded DNA (ssDNA) contents (d), and apoptosis (e) were assessed. (f, g) Annexin V FACS assays were performed. The values are represented as mean ± SD. ∗*P* < 0.05 vs. “Ctrl” group. ^#^*P* < 0.05 vs. cells treated with H_2_O_2_ only.

H_2_O_2_ stimulated cell death and apoptosis in primary murine chondrocytes. Therefore, whether OI could protect chondrocytes from H_2_O_2_-induced cytotoxicity was explored. As illustrated in Figure 2, H_2_O_2_ (300 *μ*M) treatment of primary murine chondrocytes led to a robust viability reduction (Figure 2(a)) and an increase in cell death (Figure 2(b)), which were potently ameliorated by OI treatment (Figures 2(a) and 2(b)). Additionally, our results demonstrated that H_2_O_2_ activated apoptosis activities, evidenced by cleavage of caspase-3 (Figure 2(c)), accumulation of single-stranded DNA (ssDNA), and elevation of the TUNEL staining and of Annexin V ratio (Figure 2(d)). Significantly, H_2_O_2_-induced apoptosis activation was largely inhibited by OI treatment (Figures 2(c) and 2(d)). Notably, OI treatment alone exerted no significant effect on chondrocytes. Collectively, the results showed that OI dramatically attenuated the cytotoxicity and apoptosis caused by H_2_O_2_ in primary murine chondrocytes.

### 3.3. OI Alleviated H_2_O_2_-Stimulated Oxidative Injury and Programmed Necrosis in Primary Murine Chondrocytes

**Figure 3:**
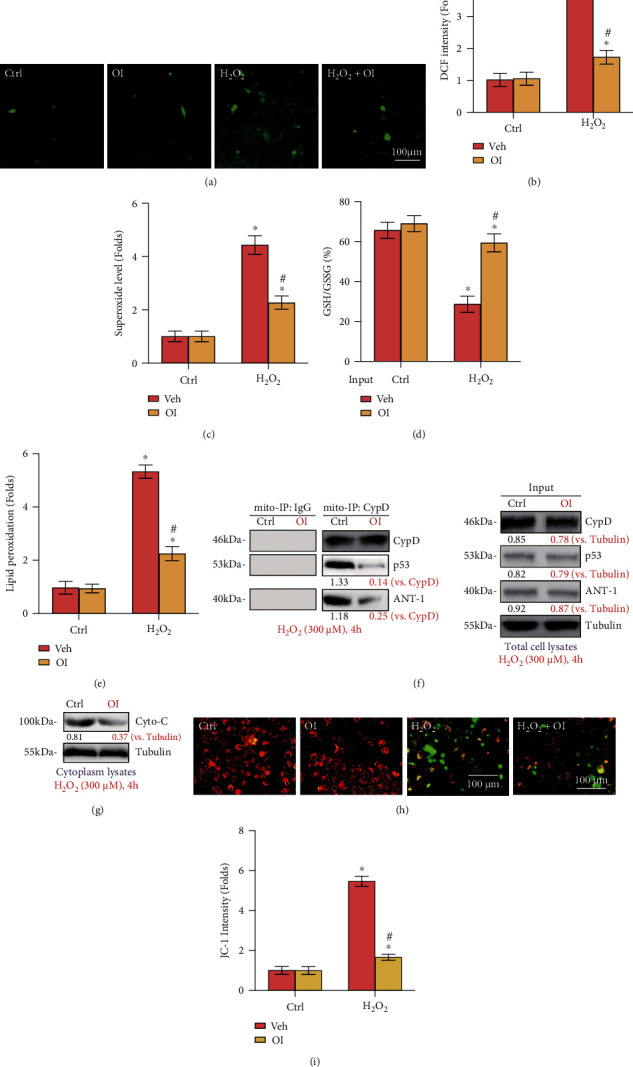
OI alleviated H_2_O_2_-stimulated oxidative injury and programmed necrosis in primary murine chondrocytes. Primary murine chondrocytes were added with OI (25 *μ*M) or vehicle control (“Veh”), followed by H_2_O_2_ (300 *μ*M) stimulation. ROS production (a, b), superoxide level (c), the GSH/GSSG ratio (d), and lipid peroxidation (e) were assessed using the corresponding assays. Mitochondrial CypD-p53-ANT-1 association (“Mito-IP”, f), cytochrome C (“Cyto-C”) release (g, testing cytosolic proteins), and mitochondrial depolarization (h, i) were measured. The values are represented as mean ± SD. ∗*P* < 0.05 vs. “Ctrl” group. ^#^*P* < 0.05 vs. cells treated with H_2_O_2_ only.

Emerging evidence has revealed that H_2_O_2_ triggers profound ROS generation and oxidative stress in chondrocytes, resulting in subsequent cell death and apoptosis. In contrast, suppression of oxidative injury can protect chondrocytes from H_2_O_2_. The above results demonstrated that OI effectively alleviated H_2_O_2_-induced cytotoxicity and apoptosis in primary murine chondrocytes; thus, its underlying effect on H_2_O_2_-induced ROS production and oxidative injury was tested. Our results showed that H_2_O_2_ caused significant oxidative injury, evidenced by ROS generation (DCF-DA intensity increase, Figures 3(a) and 3(b)), superoxide accumulation (Figure 3(c)), GSH/GSSG ratio decrease (Figure 3(d)), and lipid peroxidation (Figure 3(e)). These actions were largely attenuated with OI treatment.

Additionally, our results revealed that H_2_O_2_ treatment activated programmed necrosis in primary murine chondrocytes. Mitochondrial immunoprecipitation (Mito-IP) assay results demonstrated that H_2_O_2_ induced immunoprecipitation of p53 with CypD and ANT-1, two major components of the mitochondrial permeability transition pore (mPTP) [27], while the expression of p53, CypD and ANT-1 remained unchanged (Figure 3(f), “Input”). In addition, cytosolic cytochrome C (Cyto-C) release (Figure 3(g)) and mitochondrial depolarization (Figures 3(h) and 3(i)) were detected in H_2_O_2_-stimulated chondrocytes. Remarkably, treatment with OI potently mitigated H_2_O_2_-induced programmed necrosis activation. Collectively, these data revealed that OI inhibited oxidative stress and programmed necrosis stimulated by H_2_O_2_ in chondrocytes.

### 3.4. Nrf2 Activation Was Required for OI-Induced Chondrocyte Protection from H_2_O_2_

**Figure 4:**
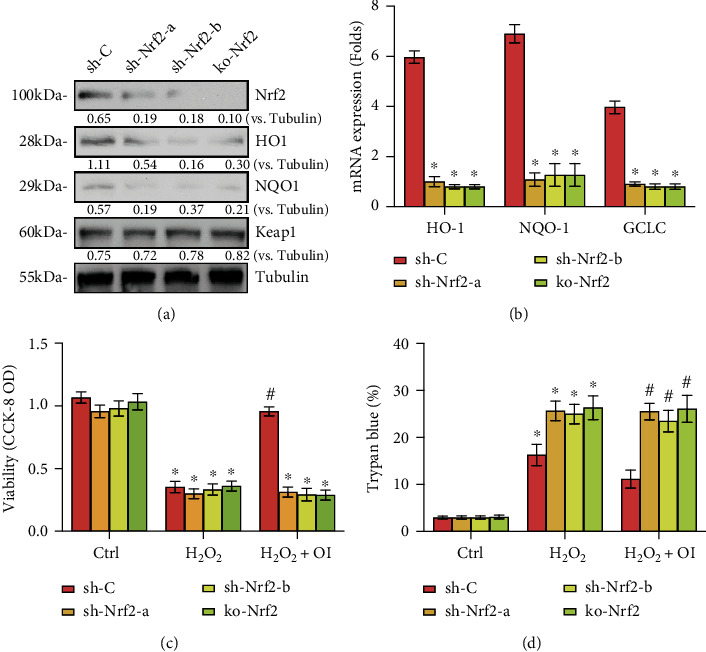
Nrf2 activation was required for OI-induced chondrocyte protection from H_2_O_2_. (a, b) Stable chondrocytes with the indicated Nrf2 shRNA (sh-Nrf2-a; sh-Nrf2-b) or ko-Nrf2, as well as control cells with scramble control shRNA (sh-C), were established, and the expression of the listed genes was examined. Chondrocytes were added with OI (25 *μ*M), followed by H_2_O_2_ (300 *μ*M) stimulation. Cell viability and apoptosis were detected using CCK-8 (c) and Trypan blue assays (d), respectively. Data are represented as mean ± SD. ∗*P* < 0.05 vs. “Ctrl” group. ^#^*P* < 0.05 vs. “sh-C” cells.

To confirm whether Nrf2 activation primarily contributes to the protective effect of OI in chondrocytes treated with H_2_O_2_, Nrf2 was silenced or depleted. Two lentiviral Nrf2 shRNAs with nonoverlapping sequences were individually transfected into proliferating primary murine chondrocytes, and two stable cells were employed after puromycin selection (sh-Nrf2-a and sh-Nrf2-b). In addition, stable chondrocytes with the CRISPR/Cas9-Nrf2-KO-GFP construct were established (ko-Nrf2). And results of Figures 4(a) and 4(b) showed that OI-induced Nrf2 protein stabilization as well as the mRNA and protein levels of *HO1*, *NQO1*, and GCLC were almost completely blocked with Nrf2 silenced or depleted in chondrocytes.

Functionally, sh-Nrf2 and ko-Nrf2 chondrocytes were more vulnerable to H_2_O_2_ stimulation, showing reduced viability (Figure 4(c)) and enhanced cell death (Figure 4(d)). However, OI was ineffective in ameliorating the reduced viability and cell death stimulated by H_2_O_2_, indicating that the cytoprotective effect of OI was abrogated by Nrf2 silenced or depleted. These findings proved that Nrf2 cascade was involved in the cellular protection of OI in H_2_O_2_-stimulated chondrocytes.

### 3.5. Keap1 Knockout Abolished OI-Induced Cytoprotection from H_2_O_2_

**Figure 5:**
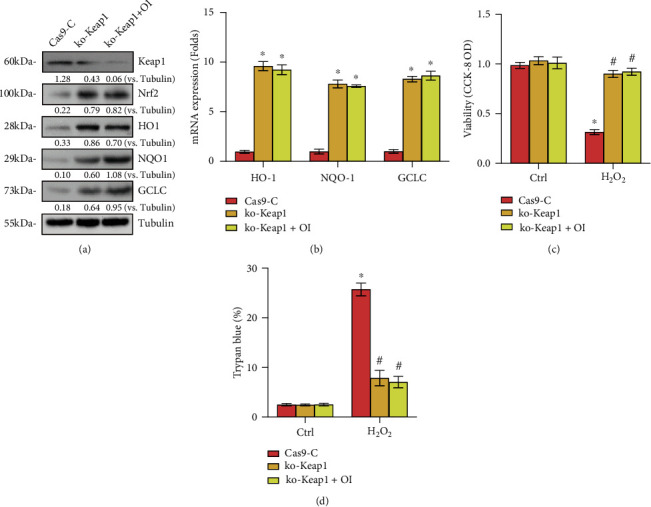
Keap1 knockout abolishes OI-induced cytoprotection from H_2_O_2_. Stable chondrocytes with the CRISPR/Cas9-Keap1-KO-GFP construct (ko-Keap1) or the CRISPR/Cas9 control vector (“Cas9-C”) were established, and the expression of the listed genes was tested (a, b). Following treatment with OI (25 *μ*M), chondrocytes were added by H_2_O_2_ (300 *μ*M). Cell viability and apoptosis were assessed using CCK-8 (c) and Trypan blue assays (d), respectively. The values are represented as mean ± SD. ∗*P* < 0.05 vs. “Ctrl” group. ^#^*P* < 0.05 vs. “Cas9-C” cells.

It has been demonstrated that OI modifies Keap1, leading to separation of Nrf2 from Keap1 and activation of Nrf2 signaling [13]. Based on this, we predicted that the cytoprotective effect of OI on chondrocytes would be nullified by Keap1 depletion. To examine this prediction, a CRISPR/Cas9-KO-Keap1 construct was used to transfect primary murine chondrocytes, and stable chondrocytes were established (ko-Keap1). As illustrated in Figures 5(a) and 5(b), ko-Keap1 led to depletion of Keap1 protein and stabilization of Nrf2 protein, as well as potently upregulated levels of HO1, NQO1, and GCLC. Furthermore, viability decrease (Figure 5(c)) and apoptosis (Figure 5(d)) resulting from H_2_O_2_ exposure were mostly attenuated by ko-Keap1 in primary murine chondrocytes. Notably, ko-Keap1 cells treated with OI failed to boost the Nrf2 pathway and offered no cytoprotection against H_2_O_2_. The findings indicated that activating Nrf2 pathway was required for the protective effect of OI against H_2_O_2_ in chondrocytes.

### 3.6. OI Inhibited OA Progression in DMM Mouse Model

**Figure 6:**
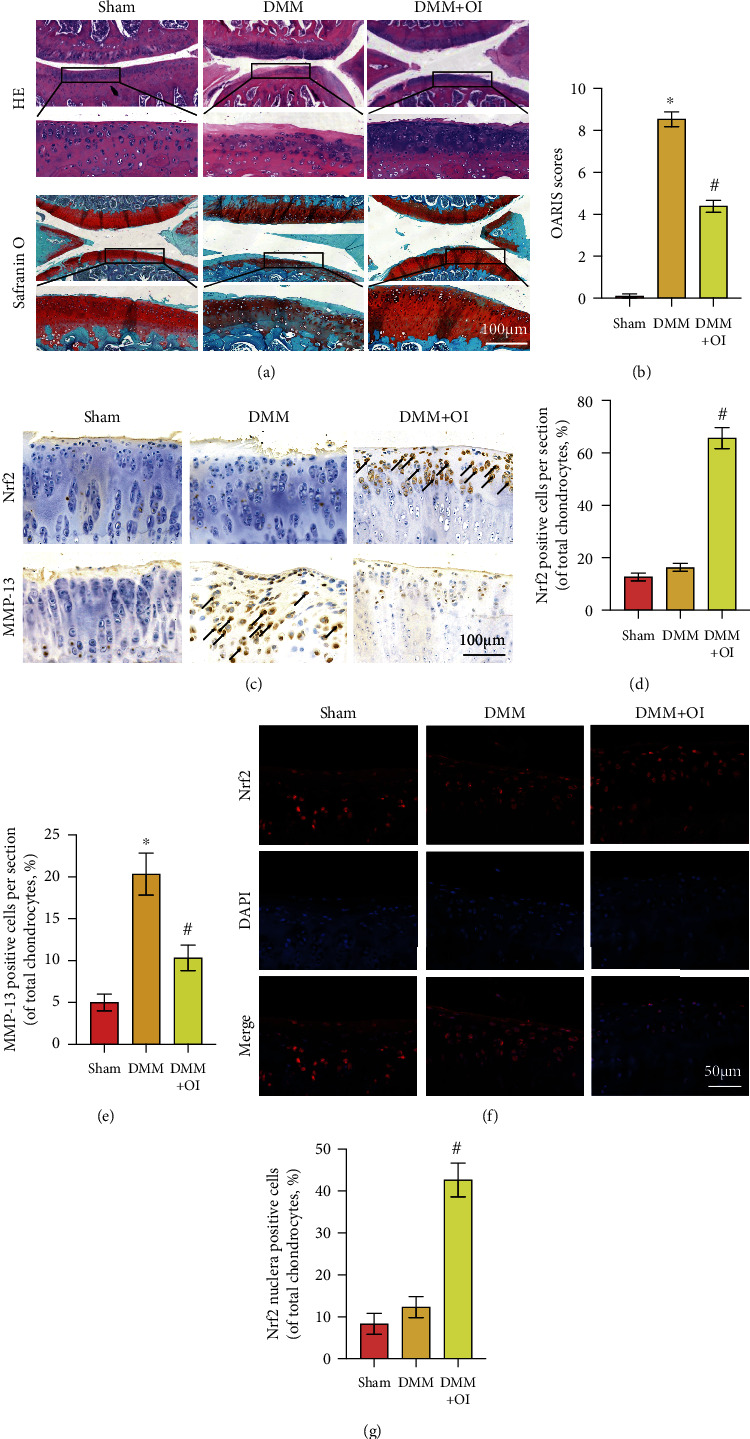
OI inhibited OA progression in DMM mouse model. (a) Histological analysis was assessed by HE and safranin O staining. (b) The OARSI scores were recorded. (c) The expressions of Nrf2 and MMP-13 in cartilage samples were detected by immunohistochemistry staining. (d, e) Quantitative analysis of positive Nrf2 and MMP-13 expressions in each section. (f) Immunofluorescence staining of Nrf2 in the cartilage samples. (g) Nrf2-positive cells in total chondrocytes of the cartilage in different groups. Data are represented as mean ± SD. ∗*P* < 0.05 vs. “Sham” group. ^#^*P* < 0.05 vs. “DMM” group.

A mouse DMM model was established to determine the effect of OI on OA progression. As shown by hematoxylin and eosin (HE) staining and safranin O staining (Figure 6(a)), the OA group revealed more severe cartilage erosion, more proteoglycan loss, and fewer cells than the control and OI groups. Quantitative analysis using Osteoarthritis Research Society International (OARSI) scores (Figure 6(b)) was in line with the findings of HE and safranin O staining. The OARSI scores of the OI group was distinctly lower than that of the OA group. Furthermore, immunohistochemical staining of the cartilage tissues for Nrf2 and MMP13 was performed. As shown in Figures 6(c)–6(e), higher level of MMP13 was observed in the OA group than in the control group, whereas the level of MMP13 was decreased in the OI group. Notably, the OI group showed a significantly higher number of Nrf2 nuclear-positive chondrocytes than the control and OA groups. In addition, to investigate whether Nrf2 was involved in OI-induced protection of OA progression, Nrf2 in the cartilage were measured by immunofluorescence staining. Results of Figure 6(f) showed that Nrf2 was mostly concentrated in cytosol in the sham and DMM groups, whereas the DMM + OI group showed significantly increased Nrf2 nuclear-positive chondrocytes. Quantitative analysis (Figure 6(g)) also confirmed that. Collectively, these data indicated that OI ameliorated OA progression and that the Nrf2 cascade was involved in this process.

## 4. Discussion

It has been widely shown that the Nrf2-associated signaling pathway suppresses ROS generation and subsequently offers vital protection against oxidative damage. Once activated, Nrf2 separates from Keap1, enters the nucleus, and associates with AREs to initiate increase of some antioxidant enzymes and detoxifying genes, thereby exerting ROS scavenging and antioxidant functions [6, 10, 28]. Accumulating evidence has indicated that oxidative stress contributes greatly to the progression of OA. In contrast, radical scavengers and antioxidant agents are considered therapeutic strategies to protect chondrocytes against oxidative injury to attenuate OA progression [29]. Therefore, the Nrf2 pathway may be a promising target to treat OA.

Some previous studies have focused on effect of OI against oxidative injury via Nrf2 pathway in various cells. For example, Zheng et al. demonstrated that OI protected osteoblasts against H_2_O_2_-induced oxidative injury by Nrf2 activation [18]. Liu et al. revealed that Keap1-Nrf2 activation by OI exerted protection of neuronal cells from H_2_O_2_ [17]. Furthermore, OI activated Keap1-Nrf2 pathway to protect human umbilical vein endothelial cells against high glucose [15]. Therefore, we hypothesized that OI could protect chondrocytes against oxidative injury via activating Nrf2 pathway. In the current study, our findings indicated that OI boosted Nrf2 pathway, resulting in stabilization and nuclear translocation of Nrf2 protein, as well as upregulation of the levels of Nrf2-target genes (*HO-1*, *NQO1*, and *GCLC*) in chondrocytes. Importantly, as shown in the subsequent studies, treatment of OI potently reduced H_2_O_2_-stimulated ROS generation, oxidative stress, superoxide, lipid peroxidation, and DNA damage, revealing that treatment with OI potently inhibited H_2_O_2_-induced cell death and apoptosis in primary murine chondrocytes.

However, even several studies revealed that OI could protect different cells against oxidative stress, few reports focused on *in vivo* experiments. To investigate whether OI could protect against OA progression, the DMM mouse models were established in the current study. As expected in the DMM mice, the treatment of OI effectively attenuated ECM degradation and reduced the OARSI scores. Notably, a significantly higher number of Nrf2 nuclear-positive chondrocytes was observed in the OI-treated DMM groups, showing that OI administration ameliorated the OA progression via activation of Nrf2 in the DMM model, which was consistent with the *in vitro* results. These data offered evidence that OI could protect chondrocytes and inhibit OA progression via the Nrf2 signaling pathway. Besides, matrix metalloproteinases (MMPs) mainly consist of aggrecan and collagen II and are closely associated with the cartilage ECM degradation [30]. MMP-13, the main components of MMPs, plays an essential role in OA progression through preferentially decreasing collagen II. In our study, OI reduced production of MMP-13 via activation of Nrf2 pathway, so as to exert the anti-inflammatory effects on OA progression.

Furthermore, it has been reported that the mPTP consists of at least three main proteins (VDAC, ANT-1, and CyPD) and plays a pivotal role in cell apoptosis and programmed necrosis [27, 31, 32]. Once stimulated by H_2_O_2_, p53 translocates to mitochondria and binds to CypD and ANT-1. Then, the Cyp-D-p53-ANT-1 complex results in mitochondrial depolarization, mPTP opening, and cytochrome C release, subsequently inducing programmed necrosis. In this study, we demonstrated that OI dramatically suppressed H_2_O_2_-induced programmed necrosis in primary murine chondrocytes. Programmed necrosis, together with apoptosis, might explain the potential cellular protective effect of OI against H_2_O_2_ in chondrocytes.

Importantly, our results showed that OI-induced cytoprotection in H_2_O_2_-treated chondrocytes was almost blocked by Nrf2 silencing or depletion, indicating that Nrf2 mediates OI-induced chondrocyte protection from H_2_O_2_. Furthermore, H_2_O_2_-induced viability reduction and apoptosis were mostly ameliorated by ko-Keap1 in primary murine chondrocytes, and OI failed to activate the Nrf2 signaling cascade and offered no cytoprotection against H_2_O_2_ in ko-Keap1 chondrocytes. These results suggested that Keap1-Nrf2 activation mediated the OI-induced cytoprotection against H_2_O_2_ in primary murine chondrocytes.

The antioxidant and antiapoptotic effects of Nrf2 activation were examined in our study, which was in line with the previous results [33–36]. For example, Li et al. revealed that deltamethrin inhibited Nrf2/TLR4 signaling pathway to induce inflammation and apoptosis in cerebrums [33]. Another research showed that deltamethrin caused liver fibrosis through inhibition of Nrf2 expression and boost of NF-*κ*B/TNF-*α* and TGF-*β*1/Smad3 pathway [34]. Besides, Lv et al. demonstrated that sulforaphane protected potassium dichromate-induced lung toxicity via activating Nrf2 and Akt/GSK-3b/Fyn pathway [35]. What is more, Yang et al. indicated that potassium dichromate activated Sesn2/Nrf2 pathway to cause heart dysfunction [36]. These studies revealed that other molecules, such as TLR4, NF-*κ*B/TNF-*α*, TGF-*β*1/Smad3, Akt/GSK-3b/Fyn, and Sesn2, were also involved in the cytoprotection against oxidative damage. However, our experiment only examined the effect of Nrf2 pathway against oxidative damage. Hence, more experiments should be performed to reveal the signaling pathway of OI in chondrocyte protection in the future.

## 5. Conclusions

**Figure 7:**
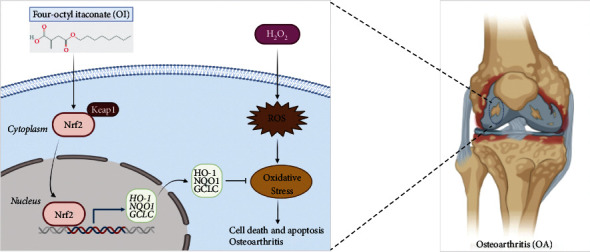
Schematic of the chondroprotective effect of OI via the Nrf2 pathway. OI protects chondrocytes against H_2_O_2_-induced oxidative stress by activating Nrf2, which translocates into the nucleus to increase the transcription and expression of Nrf2-dependent antioxidant proteins (HO1, NQO1, and GCLC), and then attenuates OA progression.

Our results showed that OI could protect primary murine chondrocytes against H_2_O_2_-induced oxidative injury and cytotoxicity via the activation of the Keap1-Nrf2 signaling pathway. Meanwhile, OI may retard OA progression in mice (Figure 7). Hence, our study provided evidence that OI might serve as a novel strategy to treat OA in clinic.

## Data Availability

The data used to support the findings of this study are included within the article.
